# Postnatal management of preterm infants with spinal muscular atrophy: experience from German newborn screening

**DOI:** 10.1186/s13023-024-03362-z

**Published:** 2024-09-26

**Authors:** Regina Trollmann, Jessika Johannsen, Katharina Vill, Cornelia Köhler, Andreas Hahn, Sabine Illsinger, Astrid Pechmann, Maja von der Hagen, Wolfgang Müller-Felber

**Affiliations:** 1 Division of Pediatric Neurology, Department of Pediatrics, Friedrich-Alexander-University of Erlangen-Nürnberg, Loschgestr. 15, 91054 Erlangen, Germany; 2 Department of Pediatrics, University Medical Center Hamburg-Eppendorf, Hamburg, Germany; 3 Department of Pediatric Neurology and Developmental Medicine, Dr. von Hauner Children’s Hospital, Ludwig Maximilians University Hospital, Ludwig Maximilians University, Munich, Germany; 4 St. Josef-Hospital, Universitätsklinik für Kinder- und Jugendmedizin, Abteilung für Neuropädiatrie und Sozialpädiatrie, Ruhr-Universität Bochum, Bochum, Germany; 5 Department of Child Neurology, Justus-Liebig University, Giessen, Germany; 6 Hannover Medical School, Clinic for Pediatric Kidney-, Liver- and Metabolic Diseases, Hannover, Germany; 7 Department of Neuropediatrics and Muscle Disorders, Medical Center, Faculty of Medicine, University of Freiburg, Freiburg, Germany; 8 Abteilung Neuropädiatrie, Medizinische Fakultät Carl Gustav Carus, Technische Universität Dresden, Dresden, Germany

**Keywords:** 5q-SMA, Preterm infants, Spinal muscular atrophy, Newborn screening, SMA treatment, Onasemnogene Abeparvovec, Nusinersen, Risdiplam

## Abstract

**Background:**

The introduction of newborn screening (NBS) for spinal muscular atrophy (SMA) has increased the early diagnosis of 5q-associated SMA in presymptomatic and symptomatic preterm infants. National and international recommendations for treating preterms and newborns < 38 weeks of gestational age are unavailable. Our retrospective multicentre study aimed to evaluate the postnatal clinical course of preterm infants with 5q-associated SMA diagnosed since the implementation of NBS in Germany in 2021 and to summarize the German experience regarding the decision-making process for available treatment regimens for preterm infants with ≤ 3 survival of motor neuron 2 (*SMN2*) copies.

**Results:**

Twelve preterm infants with 5q-associated SMA and a mean gestational age of 34.0 weeks (range: 26.1–36.8) and birth weight of 2022 g (range: 645–3370) were reported from 8/20 German SMA NBS follow-up centers using a pseudonymized questionnaire. Confirmatory diagnosis, including *SMN2* copy number, was completed on average on postnatal day 13. All patients had a biallelic deletion of exon 7 or exons 7 and 8 of the survival of motor neuron 1 (*SMN1*) gene, with SMN2 copy numbers of two in 10 patients and three in two patients. The neonatal course was complicated by respiratory distress due to prematurity (*n* = 2), sepsis (*n* = 2), and jaundice (*n* = 2). At birth, 11 preterm infants (91.6%) were presymptomatic. However, the neurological status of one patient deteriorated at five weeks of age (postconceptional age of 41.8 weeks) prior to the start of treatment. Disease-modifying treatments were initiated in all patients at a mean postconceptional age of 38.8 weeks, with the majority receiving onasemnogene abeparvovec (83.3%, including 2 patients with prior risdiplam bridge therapy). Notably, consensus among participating experts from German neuromuscular centers resulted in 83.3% of patients receiving disease-modifying treatment at term.

**Conclusions:**

Premature infants with SMA require interdisciplinary care in close collaboration with the neuromuscular center. SMA NBS facilitates early initiation of disease-modifying therapy, ideally during the presymptomatic phase, which significantly influences the prognosis of the newborn.

## Background

Recessively inherited 5q-associated spinal muscular atrophy (SMA) is one of the most common monogenic causes of death in infancy due to progressive muscular atrophy, weakness, and bulbar dysfunctions [1]. Biallelic loss-of-function pathologic variants in the survival motor of neuron 1 (*SMN1*) gene result in reduced survival of motor neuron (SMN) protein and progressive degeneration of motor neurons in the spinal cord and caudal brain stem [2, 3]. In more than 95% of the patients, SMN deficiency is caused by the homozygous deletion of exon 7 or exons 7 and 8 of the *SMN1* gene (5q11.2-q13.3). The *SMN1* gene sequence differs by only five nucleotides from the paralogous survival of motor neuron 2 (*SMN2*) gene, whose copy number varies from 1 to 6 (0 = lethal). Due to a splicing defect only 10–20% of *SMN2* transcripts include exon 7. Disease severity is related to the number of *SMN2* gene copies [1, 4]. Patients who develop clinical symptoms in early infancy usually have two or three *SMN2* gene copies. The clinical phenotype of the disease is classified according to the achievement of verticalization and the degree of mobility (SMA type 1–4). Based on data from newborn screening (NBS) for SMA in Germany, the estimated incidence of autosomal recessive 5q-SMA is 1 in 7500 newborns [5].

The clinical course and phenotype of this life-limiting disease have changed dramatically since the availability of disease-modifying treatments [4]. The first disease-modifying drug, the antisense oligonucleotide nusinersen, which acts as an *SMN2* splicing modifier by binding to an intronic splice silencing site N1 in intron 7 of the *SMN2* pre-messenger RNA, was approved in 2017 to treat all types of 5q-SMA, including presymptomatic neonates. Its lifelong intrathecal use is recommended. Onasemnogene abeparvovec (OA), which involves the adeno-associated virus (AAV) 9-mediated transfer of a new copy of the *SMN1* gene to the host, was approved in 2020 as the first gene replacement therapy for intravenously treating patients with 5q-SMA with biallelic *SMN1* mutations and clinically diagnosed with SMA type 1 or patients with ≤ 3 *SMN2* gene copies (including presymptomatic newborns). The *SMN2* splicing modifier risdiplam, which acts by binding to exonic splicing enhancer 2 (in exon 7), has been available for orally treating all types of SMA since 2021. In 2023 risdiplam was approved for treating presymptomatic and symptomatic neonates [4, 6]. Early treatment with any of the approved drugs is of crucial prognostic value, especially in newborns with ≤ 3 *SMN2* gene copies, since it significantly modifies acute and longer-term disease outcomes [4, 7, 8]. Therefore, the early identification of newborns with SMA is critical in terms of prognosis. In Germany, 5q-SMA genetic screening has been included in general NBS since 2021. It allows the detection of a homozygous deletion of exon 7 in the *SMN1* gene with 95% sensitivity. A definitive diagnosis is confirmed by polymerase chain reaction, including *SMN2* copy number analysis. Recent results from SMA NBS in Germany confirm its feasibility and high specificity [9].

The successful introduction of SMA NBS has increased the early diagnosis of 5q-associated SMA in presymptomatic and symptomatic preterm infants (≤ 37 weeks of gestational age [GA]). However, national and international recommendations for treating preterms and newborns < 38 weeks GA are unavailable. Moreover, none of the available disease-modifying treatments (onasemnogene abeparvovec, nusinersen, and risdiplam) are approved for treating preterm infants.

Controlled trials did not include newborns with a GA < 38 weeks [4, 7, 10]. Therefore, given the progressive course of the disease and the potential risks of novel treatments, the optimal time to treat infants born preterm remains an open question and represents a complex therapeutic challenge. The US Food and Drug Administration (FDA) explicitly did not recommend treating preterms with onasemnogene abeparvovec before reaching full-term GA because of the potential adverse long-term effects of the required accompanying corticosteroid treatment [11].

Our retrospective multicentre study aimed to evaluate the postnatal clinical course of preterm infants with 5q-associated SMA diagnosed since the implementation of NBS in Germany in 2021 and to summarize the German experience regarding the decision-making process for the available treatments for preterm infants with ≤ 3 *SMN2* gene copies.

## Methods

Pediatric neurology clinics and treatment centers in Germany that the German Muscular Dystrophy Society selected as SMA NBS follow-up centers (https://dgm-behandlungszentren.org; *N* = 20) were asked to participate in this retrospective multicentre study on the German experience with premature infants with SMA identified by the German SMA NBS. After confirmation of participation, the centers were asked to complete a pseudonymized data questionnaire that included clinical data, genetic findings, and the type and time of disease-modifying treatment. This study was approved by the local ethics committee of the Friedrich-Alexander University of Erlangen-Nuremberg and complied with guidelines on human experimentation. The inclusion criteria were (1) GA < 38 weeks of gestation, (2) positive NBS screening result between October 2021 (when the nationwide SMA NBS was introduced) and March 2024. This study also included one patient diagnosed in April 2021 during the pilot phase of the German SMA NBS.

### Statistics

The data were described using descriptive statistics and analyzed using GraphPad Prism v9.5.1 (La Jolla, USA).

## Results

### Diagnostic procedures

Twelve preterm newborns with positive SMA NBS born between October 1, 2021, and March 30, 2024 (including one diagnosed in April 2021 during the German Pilot SMA-NBS project), were reported from eight centers (Table 1). A homozygous deletion in the *SMN1* gene was confirmed in all cases. The *SMN2* copy number was two (*n* = 10) or three (*n* = 2). Their mean GA was 34.0 weeks (range: 26.1–36.8).

**Table 1:** The characteristics of the premature infants with SMA detected by the German nationwide SMA NBS

Number of confirmed preterm infants with SMA	12 (7 females)
Gestational age (at birth) in weeks, mean (min–max)	34.0 (26.1–36.8)
Birth weight (grams), mean (min–max)	2022 (645–3370)
Age at confirmatory diagnostics (days), mean (min–max)	9.4 (7.0–13.0)
Age at genetic confirmation (days), mean (min–max)	13.2 (10.0–17.0)
Mean PCA at genetic confirmation (weeks), mean (min–max)	35.9 (28.1–38.7)
*SMN2* copy number (*n*)	
Two	10
Three	2

Confirmatory diagnostics were performed at a mean age of 9.4 days (range: 7.0–13.0). Genetic confirmation of the diagnosis, including determination of the *SMN2* copy number, was completed at a mean postnatal age of 13.2 days (range: 10.0–17.0; Table 1). Consultation at a neuromuscular center was available for all patients, usually within two working days. However, there were longer intervals for three cases with GAs of 26, 30, and 31 weeks, who were initially treated in neonatal intensive care at their home hospital until they were close to term. In these cases, confirmatory diagnostics were initiated by the neonatologist in close collaboration with the neuromuscular center (SMA screening center). Parents were counseled by an expert from the relevant neuromuscular center after confirmation of the diagnosis, and local neonatologists and pediatric neurologists regularly monitored the neurological findings in consultation with the neuromuscular center. All cases were transferred to the appropriate hospital experienced in all currently available SMA-specific treatments as soon as they met the indication criteria for these treatments.

### Prematurity characteristics

Seven patients were born via spontaneous vaginal delivery, and five by cesarean section. Two patients born at 33 weeks of GA were twins. Maternal risk factors were present in two cases (HELLP syndrome and maternal drug abuse). The mean birth weight was 2022 g (range: 645–3370). Two cases were born with intrauterine growth retardation (IUGR). The mean five-minute Apgar vitality index was 8.9 (6.0–10.0), and the mean umbilical artery pH value was 7.27 (6.95–7.36; *n* = 10). Characteristic problems of prematurity presented at birth and/or developed in the early postnatal period in three cases with GAs of 26, 31, and 35 weeks (Table 2). The neonatal course was complicated by respiratory distress due to prematurity (*n* = 2), sepsis (early-onset [*n* = 1] or late-onset [*n* = 1]), or neonatal hyperbilirubinemia (*n* = 2). Neonatal late-onset sepsis and meningitis developed at postnatal day 12 in one neonate born at 35 weeks GA with IUGR (birth weight: 1720 g). One patient (GA 31 weeks) received primary mechanical ventilation for 22 days due to neonatal respiratory distress syndrome. Two other cases (GA 26 and 36 weeks) required respiratory support with high-flow oxygen therapy for 67 and 2 days, respectively. One patient (GA 26) was diagnosed with bronchopulmonary dysplasia. The mean hospital stay length was 34.2 days (range: 2–91). Cardiac abnormalities were diagnosed in two patients: one had patent foramen ovale with left-to-right shunt without hemodynamic problems, and the other had patent ductus arteriosus with left-to-right shunt, tricuspid valve insufficiency, and an anomaly of the pulmonary veins (Table 2).

**Table 2:** Postnatal characteristics related to prematurity

GA (weeks)	SMN2 copy number	Pulmonary	Respiratory support	Cardiac	Infection	Others
30	2	-	-	PFO with left to right shunt	-	-
34	2	-	-	-	-	
36	2	Transient disturbances of adaptation	High flow, 2 d	-	-	-
36	3	-	-	-	-	
36	2	-	-	-	-	
31	3	ARDS PPHN (mild) missing thoracic excursion	Mechanical ventilation, 22 d	Bradycardia PDA with left to right shunt, Tricuspid valve insufficiency, anomaly of the pulmonary veins; signs of right heart strain (47th day of life)	early onset sepsis	hyperbilirubinemia
35	2	-	-	-	Late onset sepsis, meningitis	-
36	2	-	-	-	-	-
35	2	-	-	-	-	-
26	2	ARDS, BPD	High flow, 67 d	-	-	-
33	2	-	-	-	-	-
33	2	-	-	-	-	-

*Abbreviations* GA, Gestational age; PCA, postconceptional age

### Neurological signs and symptoms

Eleven patients were presymptomatic at birth (91.6%). One infant presented with muscular hypotonia, weak sucking, and areflexia at birth (GA 31 + 5 weeks, 3 SMN2 copies). In this patient, an additional neuromuscular disease was suspected. Therefore, a muscle biopsy was performed at 36 weeks gestational age, demonstrating the unspecific finding of some atrophic fibers with altered sarcomere structure. In addition, a trio whole-exome sequencing was performed, which did not reveal any additional abnormalities. In two other patients, mild muscular hypotonia was assessed as a non-specific symptom associated with prematurity. Notably, another preterm infant (two *SMN2* copies) who was presymptomatic at birth developed muscular hypotonia at five weeks of postnatal age (postconceptional age [PCA] of 41.8 weeks). The brain ultrasound was normal in 11 cases (including two patients with mildly enlarged ventricles), and one had mild cystic periventricular leukomalacia.

### Treatment decision-making

After informing the parents about the available disease-modifying therapies (DMT) approved for treating SMA from full-term age, DMT were initiated in all patients. Risdiplam was only approved for neonates during the study period (2023). The majority of the neonates has received onasemnogene abeparvovec (83.3%; initial treatment, *n* = 8; preceding risdiplam bridge therapy, *n* = 2). Two patients were treated with nusinersen, and a further two patients with risdiplam (bridging therapy). The centers preferred to care for the patients in the neonatal intensive care unit of the perinatal center where they were born and to transfer them to the specified Children’s Hospital for disease-modifying treatments as soon as the necessary prerequisites were met (*n* = 2).

Close to the initiation of treatment, the mean Children’s Hospital of Philadelphia-Infant Test of Neuromuscular Disorders (CHOP-INTEND) score was 33 (23–47; *n* = 7), determined at a mean postnatal age of 40.5 days (range: 21.0–70.0) and PCA of 38.7 (range: 36.0–42.0) weeks.

Disease-modifying treatments were initiated at a mean age of 32.9 days (range: 7.0–79.0; median: 26.5); that is, at a mean PCA of 38.8 (range: 34.9–42.6; median: 39.0; Table 3). The mean time interval between confirmation of the diagnosis and the initiation of therapy was 20.7 days (range: 1–65; median: 14.5). Ten cases were treated with onasemnogene abeparvovec at a mean PCA of 39.7 weeks (range: 37.8–42.6; mean postnatal day of 42.9 (range: 24.0–79.0). Two patients were treated with nusinersen at a PCA of 38.7 and 38.1 weeks (Table 3). In two premature infants born after Risdiplam was approved for use in the neonatal period, treatment with risdiplam was initiated from day 7 of life (PCA 34.9 weeks) as a bridge to gene replacement therapy after reaching term (Table 3).

**Table 3:** Treatment decision

	Total	Onasenogene abeparvovec	Nusinersen	Risdiplam (*R*)◊ Onasenogene abeparvovec (OA)
Number of patients (*n*)	12	8	2	2
*SMN2* copy number	Two, *n* = 10 Three, *n* = 2	Two, n=6 Three, n=2	Two, *n* = 2 Three, *n* = 0	Two, *n* = 2 Three, *n* = 0
PCA at the start of treatment (weeks)	Mean: 38.8 (range: 34.9–42.6)	Mean: 39.7 (range: 37.8–42.6)	38.7; 38.1	R: 34.9; 34.9 OA: 38.9; 38.9
	Median: 39.0	Median: 39.6		
Postnatal age at the start of treatment (days)	Mean: 32.9 (range: 7–79)	Mean: 42.9 (range:24–79)	17; 21	R: 7; 7 OA: 37; 37
	Median: 26.5	Median 38.5		
Time interval between confirmation of diagnosis and the start of therapy (days)	Mean: 20.7 (range: 1–65)	Mean: 29.3 (range: 7–65)	1; 10	R: 1; 1
	Median: 14.5	Median: 25.0		

AAV titers were negative in all cases before treatment with onasemnogene abeparvovec (< 1:50). All cases treated with onasemnogene abeparvovec received prednisolone at 1 mg/kg/d according to the recommended regimen. In one case, the initiation of treatment was delayed due to late-onset sepsis and meningitis; in the other cases, there were no delays in treatment due to medical problems or delays in insurance coverage, although in one case, the decision to treat was complicated by unresolved custody issues.

During a 12-week post-treatment interval, elevated liver enzymes were reported in 4 patients, with values > 2 times the upper limit of normal in 2 patients. Peak values of GOT (mean 129 U/L) and GPT (mean 56 U/L) were measured in these patients at a mean interval of 38 and 22 days post-treatment, respectively. During the first week of OA treatment, three patients developed vomiting and/or fever. One of the infants developed thrombopenia on day 43 after OA treatment.

## Discussion

Using a multicentre, retrospective study design, we presented data on the postnatal clinical presentation of preterm infants with 5q-associated SMA and the German experience regarding the treatment decision in preterm infants with ≤ 3 *SMN2* copies since the introduction of NBS for SMA in Germany in 2021. Neonatal symptoms, especially respiratory and cardiac problems, were typical of prematurity, and 11/12 cases showed a presymptomatic course without typical SMA symptoms up to term. In one case, who was presymptomatic at birth, muscular hypotonia developed at five weeks postnatal (PCA of 41.8 weeks). Confirmatory diagnosis was completed on average at postnatal day 13. Disease-modifying treatments, preferably OA treatment, were initiated in all cases at a mean PCA of 38.8 weeks, and thus earlier than in neonates born at full-term age (German NBS data [9]).

## SMA NBS

Since the introduction of nationwide screening for SMA as part of the general NBS program, presymptomatic and symptomatic 5q-associated SMA is diagnosed increasingly in preterm infants. Here, we presented a large case series of 12 preterm infants in which NBS allowed us to make the diagnosis within an average of 13.2 days. Thus, specific counseling of the parents and treatment planning could already occur at a mean PCA of 35.9 weeks. While inpatient neonatological treatment and monitoring were primarily required in half of the cases, there were no problems with the communication of screening or confirmation diagnostic findings or with direct cooperation with the neuromuscular center.

In addition to neonatal intensive care, the only treatment available for preterm infants is symptomatic therapy according to the recommended standards of care [1]. Given the rapidly progressive nature of the disease according to the genotype [7, 10, 12], comprehensive counseling of the parents and responsible decision-making regarding the indication and optimal timing of treatment are very challenging issues. Close interdisciplinary collaboration between perinatal and neuromuscular centers is necessary, as demonstrated here by our data on preterm infants with a GA of 26–36 weeks.

The parents received comprehensive advice at the neuromuscular center about the options for treatment with disease-modifying therapies. In two cases of extremely premature birth (very low birth weight [VLBW]), the initial diagnostic consultation was conducted by the neonatologist in close cooperation with the pediatric neurologist. All centers confirmed that the challenging time before the initiation of disease-modifying therapy, which is not recommended until close to full-term age, places an enormous psychological burden on the parents and the caring team. Recent data based on a parent-related questionnaire of families with SMA newborns detected in a pilot NGS project for SMA in Germany indicated high parental psychosocial burden, uncertainty, and anxiety, emphasizing the need for comprehensive parental psychosocial support [13].

### Clinical presentation

Five of the 12 patients in our cohort required neonatal intensive care due to extreme prematurity, respiratory insufficiency due to neonatal respiratory distress syndrome, sepsis and meningitis, and transient respiratory weakness. Other typical complications of prematurity were not reported. While data on the clinical presentation of preterm infants with SMA are limited, there are some recent case reports (Table 4) demonstrating neonatal problems due to prematurity similar to our observations [14–18]. Our study, which also included extremely and very premature infants, highlights the risk of infections as a well-known complication due to prematurity.

**Table 4:** A review of published case reports of premature infants with SMA

Ref., number of pts (*N*)	GA (wks)	SMN2 copies	Respiratory symptoms	Respir. support (duration)	others	Symptoms (start of treatment)	Treatment 1 (postnatal age)	Treatment 1 PCA (weeks)	Treatment 2 (postnatal age)
Nigro et al. (2023), [15] *N* = 1	32	2	ARDS	CPAP (3 d)	Jaundice, phototherapy	Presymptomatic	Nusinersen (4 wks)	36	OA (10 wks)
Lee et al. (2021), [18] *N* = 2	34 + 1	2	no	no	Feeding problems*	Presymptomatic	OA (22 d)	37 + 2	no
	34 + 6	2	no	no	Jaundice, phototherapy	Symptomatic**	OA (37 d)	40 + 1	no
Ferrante et al. (2022), [16] *N* = 1	30	2	ARDS	CPAP (24 d)	no	Presymptomatic	Nusinersen (19 d)	32 + 5	OA (84 d)
Chiang et al. (2023), [14] *N* = 2	35 + 6	2	no	no	no	Presymptomatic	Nusinersen (n.d.)	n.d.	OA (3 wks)
	32 + 2	2	ARDS	Oxygen (4 mo)	n.d.	Presymptomatic	Nusinersen (6 wks)	38 + 2	OA (9 wks)
Lee et al. (2022), [17] *N* = 7	34 + 1 – 36 + 5	2 (*n* = 2) 3 (*n* = 3) 4 (*n* = 1) 5 (*n* = 1)	n.d.	n.d.	n.d.	Presymptomatic (*n* = 6) Symptomatic (*n* = 1; two *SMN2* copies)	2/3 SMN2 copies: OA (*n* = 5; mean: 34.4 d, range: 11–94)	2/3 SMN2 copies (mean: 40.0, range: 37.1–44.1) 4/5 SMN2 copies (4.5/5.0 mo)	Risdiplam (*n* = 1)

*Abbreviations* ARDS, acute respiratory distress syndrome; CPAP, continuous positive airway pressure; n.d., not determined; GA, gestational age; OA, onasemnogene abeparvovec

* Mild feeding problems (four-day stay in neonatal intensive care unit)

** The symptoms that began at the age of five weeks were absent reflexes, hypotonia, weakness, and decreased compound muscle action potential amplitudes

### Neurological findings and disease progression

Newborns with SMA type 1 often begin to show clinical symptoms within the first four weeks of life, followed by rapid deterioration of motor function [12]. However, data on the postnatal neurological status of preterm infants is very limited. In our case series, one infant (GA 31 weeks) with three *SMN2* copies had a symptomatic course from birth with muscular hypotonia, weak sucking, and areflexia. One of our patients with two *SMN2* copies showed early secondary deterioration of neurological status. While presymptomatic at birth, the patient developed muscular hypotonia from the postnatal age of five weeks (PCA of 41.8 weeks).

Consistent with our observation, a rapid clinical progression during the first postnatal weeks in preterm infants with two *SMN2* copies has been described in the literature (Table 4). A presymptomatic preterm infant at 34 weeks GA with two *SMN2* copies and a normal neurological examination (CHOP-INTEND score: 40/64 points) and normal compound muscle action potential (CMAP) amplitudes at 10 days of age showed a marked neurological deterioration after 37 days before treatment with OA was available, with findings of absent reflexes, hypotonia, and weakness (CHOP-INTEND score: 26/64 points) [18]. In a US cohort comprising seven preterm infants reported by Lee et al. [17], one infant born at 34 weeks gestation who was presymptomatic at 10 days of life (CHOP-INTEND score: 40/64) presented with areflexia, muscular hypotonia, and weakness (CHOP-INTEND score: 26/64) at a PCA of 40 weeks (the day before treatment). Others reported normal neurological and motor functioning of preterm infants with two or three *SMN2* copies at birth and during the first weeks of life before the initiation of treatment [14–16].

This observation is consistent with growing experimental evidence that motor neuron loss is a rapidly progressive process that precedes the clinical onset of SMA symptoms [2]. Multiple SMN functions are required for the functional maintenance of the neuromuscular unit. The motor neuron-specific functions are to modulate development and maturation (i.e., the development of dendrites and axons, axonal growth, and transport) [3]. In addition, many modifying factors are directly related to SMN expression but also independently modify neuronal networks, calcium homeostasis, and neurotransmission [3], which may partially explain the clinical observation that *SMN2* copy number alone does not predict disease severity.

Studies on the prenatal SMA mouse model [19] have shown that SMN protein levels in spinal cord tissue were low from early development, with a further rapid decrease during the first postnatal weeks. Human data is very limited. Autopsy studies on patients with SMA between 15 weeks of GA and 14 years of age, available from the Johns Hopkins SMA Biobank [2], showed extremely low prenatal SMN protein levels and undetectable SMN protein levels after birth. Interestingly, prenatal SMN protein levels in the spinal cord and brain of controls decreased dramatically within the first three months of life. These observations support that the early postnatal period may represent the optimal therapeutic window [2].

### Treatment decision-making process

Controlled clinical trials have impressively shown that neonates with SMA who begin disease-modifying therapies in its presymptomatic stage show a stronger treatment response than symptomatic neonates and young infants [4, 7, 10], indicating the need to initiate disease-modifying treatment as early as possible. It is important to emphasize that none of these studies included patients younger than term.

Disease-modifying treatments were initiated in all patients at a mean postconceptional age of 38.8 weeks, including 2 patients in whom treatment was initiated before PCA at 38 weeks. Thus, most of the centers participating in our retrospective study (87.5%) favored starting disease-modifying treatments at term (PCA of 38 weeks and above). A comparable procedure has been reported by others [14, 17]. The main reasons for this are believed to be the lack of international experience with the novel therapies in the sensitive age of preterm infants, as data on efficacy, complications, and adverse drug reactions are unavailable from the literature. There are no national or international recommendations regarding the therapeutic approach for premature infants with presymptomatic and symptomatic SMA. Specifically, the FDA does not recommend treating preterm infants with onasemnogene abeparvovec since adverse effects of concomitant treatment with corticosteroids on neurological development were suspected [11]. There is also a lack of controlled data on the experience with both nusinersen and risdiplam in preterm infants.

Based on the observation of significant neurological deterioration in a premature infant at 34 weeks GA within 37 days before uneventful treatment with OA at the PCA of 40 weeks, Lee et al. [18] recommended initiating treatment at 37 weeks post-conception due to the risk of rapid deterioration of neurological function in neonates with two *SMN2* copies. In our cohort, all cases had two or three *SMN2* copies, so all were indicated for treatment with disease-modifying therapies. As reported by others [9, 12, 17], most parents and physicians favor OA treatment (83.3%). A recent parent survey demonstrated that treatment frequency and administration method are the most commonly reported factors impacting parental treatment choice [20].

In our study, the time between confirmation of diagnosis and the initiation of treatment (mean: 20.7 days, range: 1–65) and postnatal time to treatment (mean: 32.9 days, range: 7–79) were longer than in the German national SMA NBS data (means = 14.3 and 31.9 days, respectively [9]) and from the Australian SMA NBS program [12]. However, the preterm infants in our study were markedly younger at the time of disease-modifying treatment (mean PCA: 38.8 weeks, range: 34.9–42.6) than the neonates in the German national SMA NBS (mean PCA: 42.5 weeks, range 38.5–48.7) [9].

The youngest infant in our study treated with omnasenogene abeparvovec was 37 + 3 weeks of PCA. Similarly, Lee et al. reported early treatment with omnasenogene abeparvovec in three premature infants at a PCA of 37 weeks [17, 18], indicating its feasibility. In addition, they found higher CMAP values in two premature infants than in five term infants with two *SMN2* copies, suggesting a better preserved motor neuron function before 40 weeks gestation [18]. However, the prognostic role of CMAP in preterm and term infants with SMA remains unknown [9].

Besides a limited number of case reports (Table 4) showing different treatment regimens, data on the safety and optimal GA for treating preterm infants and infants < 38 weeks GA is lacking. In our study, 33 weeks of postconceptional age was the youngest age at which children received treatment (risdiplam, *n* = 2). In several preterm infants reported in literature, especially those with two *SMN2* copies, nusinersen or risdiplam (*n* = 1) were used as a bridge to onasemnogene abeparvovec [14–17], and were already used before the PCA of 37 weeks [15, 16]. The youngest infant was treated with nusinersen at a PCA of 32 weeks [16]. The novel therapies’ feasibility and safety in individual cases (Table 4) and positive treatment effects [17], as well as rare complications, have been documented in preterm infants with SMA. Mildly elevated liver enzymes with an otherwise uneventful course of treatment have been reported in some cases.

The potential adverse effects of steroids on developmental neurological outcomes in preterm infants have been discussed in the literature, and were the reason for the FDA’s recommendation not to use treatment with OA in premature infants (7). Data are available from VLBW (< 1500 g) preterm infants with bronchopulmonary dysplasia receiving systemic treatment with dexamethasone or hydrocortisone. Therapy with dexamethasone within the first week of life was associated with an increased risk of cerebral palsy [21]. Due to potential adverse effects on cognitive development [22] and limited data, corticosteroid therapy (dexamethasone, hydrocortisone) is not recommended in preterm infants even after the age of seven days of life, except for high-risk preterm infants [23].

Necrotizing enterocolitis (NEC), a typical complication of prematurity, was observed in two term infants (41 and 40 weeks GA) treated with onasemnogene abeparvovec at 18 and 21 days postnatal age [24]. NEC was diagnosed 6 and 19 days after treatment with onasemnogene abeparvovec. One of the proposed pathogenetic factors in NEC is immunological dysregulation. The question of whether treatment with onasemnogene abeparvovec or concurrent steroids might increase the risk of NEC in premature and full-term infants cannot currently be answered based on the available data [24]. Therefore, evidence from the literature is insufficient to evaluate the safety of nusinersen, risdiplam or onasemnogene aboparvovec in preterm infants. Current data also do not permit a risk-benefit assessment of whether initiating therapy before 37 weeks of GA may improve the long-term prognosis of the patients compared to full-term GA. Further studies on the long-term course of premature infants may be helpful in this context.

### Limitations

This study had some limitations due to its retrospective design and small number of cases, which is due to the rarity of SMA in preterm infants. Evaluation of longer-term outcome of the preterm SMA infants and, in addition, a survey on parents’ psychosocial burden in the period between receipt of the screening result and treatment is currently being planned.

## Conclusions

In summary, our data confirms that SMA NBS enables early confirmation of diagnosis in preterm infants and early disease-modifying therapy, ideally in the presymptomatic stage of the disease, which is crucial for the future prognosis of the newborn.

## Data Availability

All data sets generated during and/or analyzed during this study are not publicly available but are available from the corresponding author upon reasonable request.

## Abbreviations

AAV: Adeno-associated virus
CHOP: INTEND-Children’s Hospital of Philadelphia Infant Test of Neuromuscular Disorders
CMAP: Compound muscle action potential
FDA US: Food and Drug Administration
GA: Gestational age
OA: Onasemnogene abeparvovec
IUGR: Intrauterine growth retardation
NBS: Newborn screening
NEC: Necrotizing enterocolitis
PCA: Postconceptional age
SMA: Spinal muscular atrophy
SMN: Survival motor neuron protein
SMN1: Survival of motor neuron 1
SMN2: Survival of motor neuron 2
VLBW: Very low birth weight
